# Clozapine and co-prescribed psychotropics: a short report

**DOI:** 10.1186/1745-0179-4-11

**Published:** 2008-04-25

**Authors:** Maneesh Gupta

**Affiliations:** 1 Lancashire Care NHS Foundation Trust, Parkwood Hospital, East Park Drive, Blackpool, FY3 8PW UK

## Abstract

Clozapine is the drug of choice in treatment resistant schizophrenia. It reduces hospitalizations. Patients on clozapine are often co-prescribed other psychotropics. This report looks at a sample of twenty patients on clozapine. It finds that almost two thirds were on a psychotropic along with clozapine. Eight individuals were on an antidepressant; seven on an antipsychotic and five were on co-prescribed valproate. The clinical implications are discussed and a need to look at health services involving clozapine is suggested.

## Background

Clozapine, a novel antipsychotic due to its receptor binding profile, is the drug of choice in treatment resistant schizophrenia (TRS). National Institute of Clinical Excellence (NICE) recommends an early initiation of clozapine therapy in individuals with TRS [1]. In UK, Clozapine is licensed to be used for treating schizophrenia in patients unresponsive to, or intolerant of, conventional antipsychotic drugs [2].

The costs of clozapine therapy are high but these are deemed to be cost effective, in view of the high levels of morbidity and disability in the target population, and the reduction of hospitalization [3-5].

Not much is known of the prescription of other psychotropics when a patient is receiving clozapine. Do most patients need only clozapine or do they still need co-prescribed medications for treatment of emergent symptoms or co morbid mental health problems? This report aims to shed some light on the use of co-prescribed psychotropics in a sample of patients receiving clozapine.

## Method

Patients on clozapine, amongst the caseload of a consultant psychiatrist (MG) were identified from the web site of clozapine prescription monitoring system (CPMS). All doctors who prescribe clozapine in England and patients who receive clozapine are registered by CPMS. Regular blood test results are received from CPMS. I used these test results to cross check that I had the right number of patients. All were being managed in the community.

In England, consultant psychiatrists or their deputed doctors, write a letter to the general practitioner of the patient they have seen, detailing their clinical observations, and clinical plan. This is termed a clinical letter. The most recent clinical letter from mental health services and the most recent prescription from the surgery were obtained. They were used as sources of information on the prescribed medicines (Clozapine and others). Psychotropics were identified (for the purpose of this report) as medication usually prescribed for the treatment of mental illness. Thus benzodiazepines, procyclidine and hyoscine (the latter two prescribed for alleviating side effects) were not included as psychotropics. Psychotropics other than clozapine were identified and the dose recorded. This information was tabulated.

## Findings

Thirteen of twenty patients had co-prescribed psychotropics (65%). The most common co-prescription, in this group of individuals, is of a serotonin specific reuptake inhibitor (SSRI) (8/20). Antipsychotics were also co-prescribed in 7 patients and 5 patients had been given valproate. None of the patients were on a co-prescribed depot antipsychotic injection (Table 1).

**Table 1:** Clozapine and co-prescribed medication (psychotropics and procyclidine)

S. No.	CLZ dose (mg)	SSRI (name/mg)	Antipsychotic (name/mg)	VAL (mg)	Others @	No. of Co-prescriptions
1.	600	-	-	-	-	0
2.	300	-	-	-	Pro 15	0
3.	250	-	Ris 1	1500	-	2
4.	500	Flu 40	-	600	-	2
5.	200	Flu 20	Ami 150	-	-	2
6.	450	-	-	-	Pro 15	0
7.	850	-	-	750	-	1
8.	600	Ser 100	Ris 2	-	-	2
9.	500	-	-	-	-	0
10.	800	Ser 100, Mir*	-	-	Pro 15	2
11.	600	Cit 20	Ami 800	-	Pro 10	2
12.	400	-	-	-	-	0
13.	825	Ser 200	Ami 400, CPZ 100	-	-	3
14.	350	Cit 20	Sul 800	-	-	2
15.	400	-	-	-	-	0
16.	500	-	Ami 200	-	-	1
17.	450	-	-	900	-	1
18.	250	Par 20	-	-	-	1
19.	500	-	-	-	-	0
20.	400	-	-	400	-	1

CLZ = Clozapine; SSRI = Serotonin specific reuptake inhibitor; VAL = Valproate; Flu = Fluoxetine; Ser = Sertraline; Mir = Mirtazapine; Cit = Citalopram; Par = Paroxetine; Ris = Risperidone; Ami = Amisulpride; CPZ = Chlorpromazine; Sul = Sulpiride; Pro = Procyclidine;

* Mirtazapine is not a SSRI but is included in this column for clarity and grouping as an antidepressant.

@ Others lists procyclidine, but has been excluded from the number of co-prescriptions.

Four patients were receiving concurrent procyclidine.

In all, 22 co-prescriptions were seen in these twenty individuals. There was no correlation with the dose of clozapine.

## Discussion

This short report shows that in a small sample of clozapine treated patients, co-prescribed psychotropics are very common. It is unfortunate that no published record of such an observation could be found. A study of health services involving clozapine will shed more light on this topic.

Clozapine is known to reduce hospitalizations, though this raises the cost of outpatient care and service utilisation [5]. While a detailed cost estimation of patients on clozapine needs to be undertaken, it has been suggested that conventional antipsychotics have no disadvantage in terms of symptoms and quality of life over a one-year period compared to atypical antipsychotics [6]. On the other hand the same appraisal suggested that clozapine was not significantly at advantage in terms of quality of life when compared to atypical antipsychotics. This report points to the high use of co-prescriptions and thus the continued high cost of health services for patients on clozapine.

Seven patients in this sample were receiving an antipsychotic along with clozapine. Augmentation of clozapine is a strategy with some support from open studies regarding amisulpride [7,8] and aripiprazole [9]. Risperidone [10] and sulpiride [11] have support from double blind randomized placebo controlled studies. Indeed this study sample had four patients on amisulpride, two on risperidone and one on sulpiride. One patient was however, on chlorpromazine (along with amisulpride).

Clozapine is not known to cause extrapyramidal symptoms. It is suggested as an antipsychotic that eliminates tardive dyskinesia. Procyclidine was co-prescribed in four patients (one patient receiving both amisulpride and clozapine) in this study sample. This is much higher than as reported in a study from France [12].

Depression has been known to coexist with chronic physical illnesses. It has also been shown that about 25% of patients with schizophrenia will experience at least 1 depressive episode in their lifetime [13] – the suicide rate of 10% holds true for the overall population of patients with schizophrenia, whereas the suicide rate in those without schizophrenia is 0.01% to 0.25% [14]. The use of SSRI to treat or address depression might be a reason for 40% of this sample being co-prescribed antidepressants.

One study has found encouraging results involving adjunctive SSRIs in treating the negative symptoms of schizophrenia [15]. However, a review of controlled studies concluded that studies with positive findings have primarily used fluoxetine or fluvoxamine [16,17] agents that are notorious for increasing the plasma concentrations of many antipsychotics. In contrast, the results of controlled studies with sertraline and citalopram, which have fewer propensities to cause interactions, have been negative [18,19].

Thus co-prescription could be explained by the high morbidity and mental health problems of the target population. It would be interesting to study whether the adjuvant medication is prescribed before clozapine started or after. This report is limited in its extent of background information and past history that could be elicited.

It is also food for thought as to whether clozapine is as complete and effective a treatment as we think it is, if clinicians do need to co prescribe a psychotropic. Is it treatment resistant schizophrenia that we are treating or is it co-morbid mental health difficulties that were not identified before clozapine was started.

## Competing interests

The author has accepted hospitality from various pharmaceutical companies in UK. He has accepted honoraria to speak at clinical gatherings from Astra Zeneca and Wyeth Laboratories. The author holds some shares in some Indian pharmaceutical companies.
